# Non-conductive ferromagnets based on core double-shell nanoparticles for radio-electric applications

**DOI:** 10.1186/s40064-016-2099-3

**Published:** 2016-04-22

**Authors:** Hélène Takacs, Bernard Viala, Vanessa Hermán, Jean-Hervé Tortai, Florence Duclairoir, Juvenal Alarcon Ramos, Pierre-Henri Jouneau, Hanako Okuno, Gwenolé Tallec

**Affiliations:** LETI, CEA, MINATEC Campus, 17 rue des Martyrs, 38054 Grenoble Cedex 9, France; LTM, CNRS-UJF, MINATEC Campus, 17 rue des Martyrs, 38054 Grenoble Cedex 9, France; INAC, CEA, 17 rue des Martyrs, 38054 Grenoble Cedex 9, France; INAC, Univ. Grenoble Alpes, 17 rue des Martyrs, 38054 Grenoble Cedex 9, France; Visualization Sciences Group, FEI, 3 Impasse Rudolf Diesel, 33700 Mérignac, France

**Keywords:** Core double-shell, Nanocomposites, Ferromagnetism, RF properties

## Abstract

Two fabrication schemes of magnetic metal-polymer nanocomposites films are described. The nanocomposites are made of graphene-coated cobalt nanoparticles embedded in a polystyrene matrix. Scheme 1 uses non-covalent chemistry while scheme 2 involves covalent bonding with radicals. Preservation of the net-moment of cobalt and electrical insulation are achieved by means of a core double-shell structure of cobalt–graphene–polystyrene. The graphene shell has two functions: it is a protective layer against metal core oxidation and it serves as the functionalization surface for polymer grafting as well. The polystyrene shell is used as an insulating layer between nanoparticles and improves nanoparticles dispersion inside the polystyrene matrix. The theoretical maximum volume filling ratio estimated at ~30 % is almost reached. The nanocomposites are shown to undergo percolation behavior but retain low conductivity (<1 S/m) at the highest filling ratio reached ~25 % leading to extremely low losses (10^−3^) at high frequency. Such low conductivity values are combined with large magnetization, as high as 0.9 T. Ability for radiofrequency applications is discussed in regards to the obtained magnetization.

## Background

Polymer nanocomposites are attractive materials due to the ability to tailor final properties. They consist in nanometer fillers embedded in a matrix. The properties are controlled by the filler volume fraction. Composites are classified into diluted or densely packed states (Leslie-Pelecky and Rieke 1996). With metallic fillers, the continuum percolation theory applies to the electrical conductivity (Hunt and Ewing 2009). Also dipolar-magnetism in interacting systems was reported (Varón et al. 2013).

In RF, polymer–metal nanocomposites are aimed to overcome the main obstacle of high-moment transition metals that are conductive. Recent works on nanocomposites of cobalt were reported but magnetization degradation was observed indicating that stable nanoparticles are needed (Nelo et al. 2010; Raj et al. 2014). They can be protected with a shell that must be a protection against surface oxidation and spin-quenching especially with cobalt (van Leeuwen et al. 1994). The shell thickness must be small as with core–shell structures, the maximum filling ratio dramatically decreases (Hanemann and Szabó 2010). The thinnest coating capable to protect metallic nanoparticles belongs to Hayashi with the use of graphene layers (Hayashi et al. 1996). From there, carbon-coated magnetic nanoparticles have emerged (Grass and Stark 2006).

**Fig. 1:**
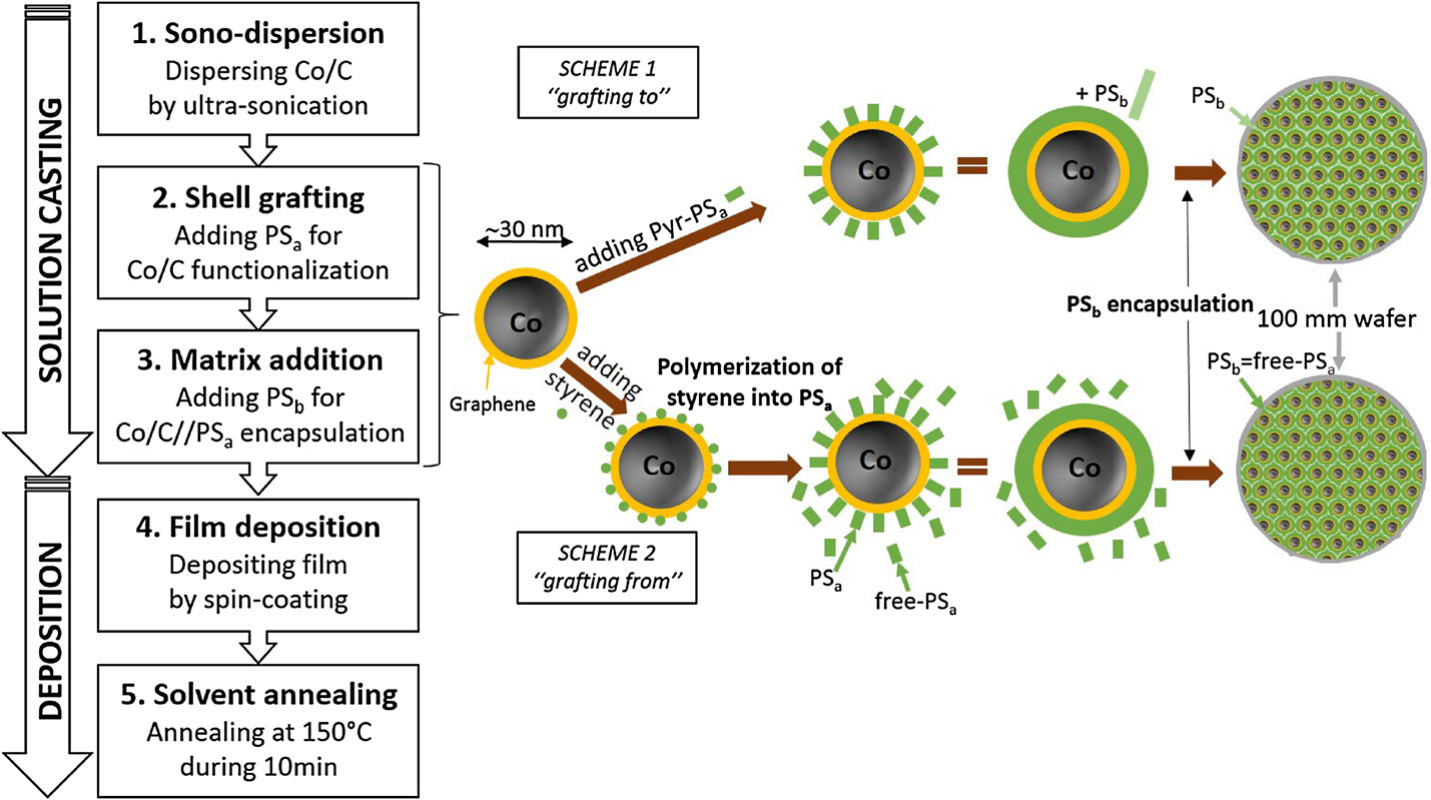
Flow chart and reaction schemes with grafting

Now, the challenge is to convert such nanopowders into a processable material. Directly mixing nanoparticles and polymers is not an option as the resulting material has poor mechanical cohesion. Hence, particles need to be strongly bound to the polymer to get a cohesive structure. It was reported with Co/C grafted elastomers in (Fuhrer et al. 2009). In this work, we address non-covalent and covalent grafting of Co/C with polystyrene (PS). The key issue is to form ultra-thin PS shells (i.e. few nm) superimposed to the existing graphene coating (i.e. conductive) for electrical insulation and avoiding that the maximum filling ratio collapses. Turbobeads^®^ (Sigma Aldrich) Co/C nanoparticles (30 nm in average) were used. A two-stage flow chart (Fig. 1) is explained with solution-casting and film-deposition. Three series of samples are considered with process variations. The nanoparticles weight fraction—and indirectly the volume fraction—was determined by thermogravimetric analysis (TGA). We report evidence of grafting between the two phases. The discussion addresses the filling limit, the conductive percolation and the impact of grafting on residual magnetization of cobalt. The tradeoff between electrical and magnetic properties is achieved.

## Methods

### Nanocomposites formulations

The overall preparation was based on sonochemistry. Two grafting schemes were explored. Scheme 1 was the radical-free option using non-covalent interactions based on electron sharing (i.e. π–π bonds). We used pyrene-terminated PS (i.e. Pyr-PS_a_) whose electronic structure is close to that of graphene. Scheme 2 used radicals to covalently graft PS–Co/C. The radicals originate from in situ polymerization of monomers of styrene. Firstly, deagglomeration of Co/C powders is prerequisite. It was performed by ultra-sonication at 100 W during 10 min, using a VCX500 Ultrasonic processor from Sonics. Note that the duration of sonication must not exceed 30 min because graphene coating deteriorates for longer sonication times.

#### Series-A (chloroform formulation: scheme 1)

The formulation used initial weight *m*_*i*_ of 300 mg of Co/C in 8 mL of chloroform (CHCl_3_) and 5 mg of Pyr-PS_a_ (polymer source) with low molecular weight *M*_*w*_ of 5.6 kg/mol aimed to form the polymeric shell (PS_a_). The intermediate product is labeled Co/C//Pyr-PS_a_-A. Subsidiary step is required to form the supporting matrix (PS_b_). To do so, 2 mL of PS (0.75 g/mL, Sigma-Aldrich) with a higher *M*_*w*_ of 35 kg/mol were added to the solution. The reaction was stopped after 30 min of sonication. Series-A is labeled Co/C//Pyr-PS_a_/PS_b_-A.

#### Series-B (anisole formulation: scheme 1)

It is a variant of series-A using anisole (C_7_H_8_O) instead of CHCl_3_ whose low boiling point *T*_*b*_ (i.e. 61.2 °C) may be a limitation for applications. Anisole (*T*_*b*_ = 153.8 °C) was aimed for spin-coating optimization. The solution casting was broader in composition with *m*_*i*_ varying from 100, 300, 500 to 700 mg. Labels are identical with replacing A by B.

#### Series-C (anisole formulation: scheme 2)

Scheme 2 is the radical option aimed to improve dispersion and cohesion. It is detailed elsewhere (Herman et al. 2015). For practical reasons, *m*_*i*_ was limited to 100 mg. The reaction time is identical (i.e. 30 min). Two sub-schemes were considered: adding Co/C at the beginning of the reaction (2a)—with a lot of radicals—or at midterm (2b)—less radicals. Thus, we expect more PS_a_ with scheme 2a. Conversely with scheme 2b more PS_b_ is awaited with a higher output of unreacted free-PS. Here, free-PS replaces additional PS_b_ (35 kg/mol) of scheme 1. Solution was precipitated in methanol to obtain a powder that was redispersed in anisole for spin-coating. Series-C is labeled Co/C//PS_a_/PS_b_-C (2a or 2b).

### Films deposition by spin-coating

Spin-coating required 1 mL of cast-formulations to deposit uniform µm-thick films on 100 mm oxidized silicon wafers. We used SPS SPIN200i-TT-PTFE spin processor. Films were solvent-annealed at 150 °C for 10 min at the end.

### Characterization tools

Nanoparticles mean hydrodynamic diameter was measured by dynamic light scattering (DLS) on a Malvern Zetasizer Nano ZS instrument. Both nanoparticles and nanocomposites were observed by high resolution transmission electron microscopy (HR-TEM) on a 200 kV Titan FEI HR-TEM. Thermal properties were characterized by modulated differential scanning calorimetry (MDSC) on a TA Q200 instrument. Nanotomography technique was carried out using Zeiss NVision 40 FIB/SEM dual beam instrument (400 slices of 3.5 nm). Thermal stability and particles weight or volume fraction were studied by TGA using a TA Instrument TGA Q500. Magnetic properties were measured by vibrating sample magnetometer (VSM) on a Microsense instrument and average film-conductivity was obtained with four-point method over 25 points on a TC1000 prober from Karl Suss. The complex permeability was measured from 10 MHz to 10 GHz by high performance coil-perturbation method using impedance analyzer (Agilent 4294A) combined with vectorial network analyzer (Agilent N5222A).

## Results and discussion

### Sedimentation time and spin-curves

Stabilized cast-solutions are required for spin-coating. Sedimentation study was conducted for Series-A after de-agglomeration, shell-grafting and matrix addition. Co/C is prone to agglomeration because of a non-null remanent magnetization at room temperature. With sonication a mixture of individual particles and clusters of ~100 nm was obtained, as measured by DLS. Such suspension sediments after only 5 min. When grafting the shell of Pyr-PS_a_ and further embedding into the matrix of PS_b_ sedimentation-time increases with 30 and 75 min, respectively, due to steric hindrance.

**Fig. 2:**
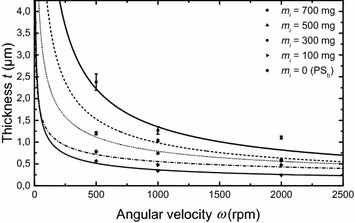
Full spin-curves established for series-B

Spin-coating is a known technique for homogeneous polymers and some emerging colloids (Rehg and Higgins 1992). However, there is no report with interacting magnetic suspensions. Before achieving reproducible films, the spinning parameters were optimized with series-B over a broad range of composition. The spin-curves are shown on Fig. 2. The error bars are representative of the radial dispersion on 4″ wafers. At first sight, the achieved thickness after solvent annealing agrees with the classical angular velocity dependence (i.e. ${ \propto }\,\upomega^{ - 0.5}$) of polymers that is remarkable here. The tradeoff for ~1 μm-thick films was found at 1000 rpm with uniformity of ~5 % indicating that radial and edge effects are weak.

### Microstructure and thermal properties

The microstructure of nanocomposites is a complex feature that required a thorough investigation.

**Fig. 3:**
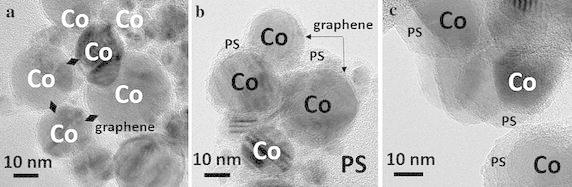
HR-TEM pictures of **a** deagglomerated Co/C particles, **b** series-B and **c** series-C 2b

HR-TEM images are presented in Fig. 3. Figure 3a shows deagglomerated Co/C nanoparticles. The graphene protective coating is well-visible with 8–10 layers (~5 nm). Series-B is presented in Fig. 3b. Graphene is unchanged, proving that scheme 1 with no radical is safe. On the contrary, series-C does not display graphene contrast anymore (Fig. 3c). Complementary observations backed with Raman analysis revealed that graphene is much thinner and partially transformed into amorphous carbon (Herman et al. 2015). Also traces of oxidized cobalt were detected by Raman spectroscopy (Herman et al. 2015). Thus, scheme 2 is more critical.

To deepen these visual observations, the glass transition temperature *T*_*g*_ was determined by MDSC. Series-B, C2a and C2b showed *T*_*g*_ at 68, 86 and 88 °C respectively which have to be compared to their own matrix. With scheme 1, PS_b_ matrix exhibit *T*_*g*_ at 62 °C while it is 75 °C with scheme 2. Thus, with metallic fillers *T*_*g*_ is higher and the difference Δ*T*_*g*_ [i.e. 6 °C (B), 11 °C (C2a) and 13 °C (C2b)] is large enough to be considered as an evidence of surface interactions (Hu et al. 2010). Higher bond dissociation energies are consistent with the assumption of covalent grafting with scheme 2. Whether covalent or non-covalent, the chemical bonds are definitely strong enough to be irreversible.

### Nanotomography and filling limits

**Fig. 4:**
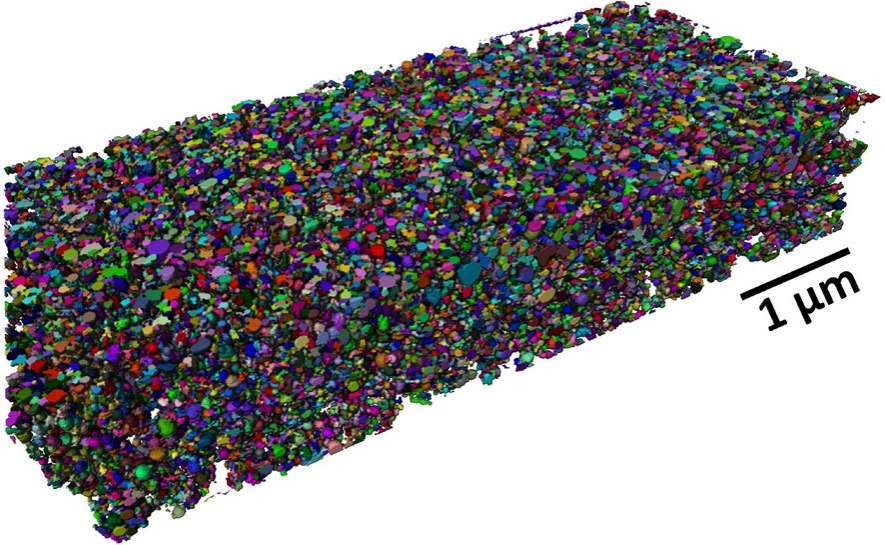
3D image of series-A. Image obtained with Avizo^®^ software

Nanotomography is an automated serial sectioning technique for high resolution 3D visualization. The technique was applied to series-A. Images were denoised and the structure was skeletonizated with Avizo^®^ software. Figure 4 shows the reconstructed volume after segmentation.

Individual particles are well visualized. The volumetric structure is made of isolated closely-spaced nanoparticles and small clusters (~100 nm) that are homogeneously dispersed.

**Fig. 5:**
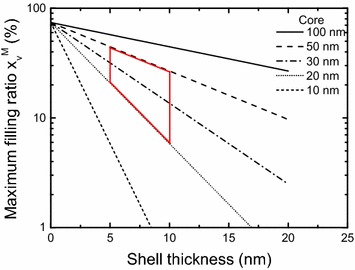
Abacus of the maximum volume ratio $x_{v}^{M}$ versus shell thickness for different core sizes

Besides weight fraction, one prefers to consider volume fraction, *x*_*v*_, for applications as physical properties in composites are often percolative. The maximum value $x_{v}^{M}$ is an important parameter. With core–shell structures, the shell-thickness is a key issue as $x_{v}^{M}$ decreases when increasing shell-thickness and when decreasing core-size (Fig. 5). For a perfect triangular lattice of monodisperse spherical particles of unprotected Co particles $x_{v}^{M}$ is 74 %. With a perfect assembly of Co(50 nm)/C(5 nm) core–shell particles $x_{v}^{M}$ would decrease to 45 % and to 27 % with core double-shell Co(50 nm)/C(5 nm)//PS_a_(5 nm). To be rigorous, we must say that it is a simple indication because actual systems deviate from assumptions of monodispersity or perfect 1:1 aspect ratio. It nevertheless gives first order upper limits achievable within this study. Extracted volume fraction from Fig. 4 is 34 %, which is roughly consistent with these estimations.

### Magnetic and electrical properties

**Fig. 6:**
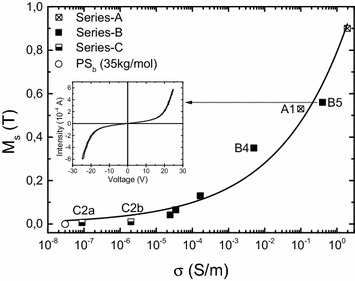
Cross-dependence of *M*_*s*_ and *σ* (series-A–C). *Inset* is I–V curve of sample B5

Figure 6 is a constitutive diagram for magnetic metal-polymer nanocomposites. It describes the cross-dependence of saturation magnetization *M*_*s*_ and conductivity *σ* that are both volume-dependent but with separate behaviors.

*M*_*s*_ is a linear function of *x*_*v*_ and reaches its maximum at $x_{v}^{M}$. Samples A1 (0.53 T, 19.3 %), B4 (0.35 T, 18.1 %) and B5 (0.56 T, 23.2 %) are close to this maximum. This confirms that the non-covalent option is safe to the magnetization. In contrast, samples C*2a* (0.007 T, 2.4 %), C*2b* (0.01T, 5.3 %) are behind as scheme 2 was not optimized and because the net-moment of Co/C has dropped ~70 % with radicals.

**Fig. 7:**
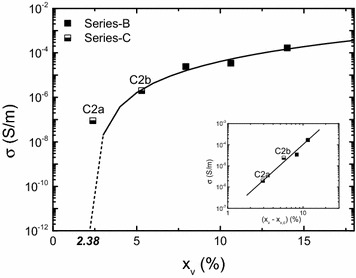
Dependence of *σ* on *x*_*v*_ (series-B, C). *Inset* is a log–log plot

The picture is different with *σ* (Fig. 7). Generally, in an ideal 3D percolative system, *σ* is the form of $(x_{v} {-}x_{v,c} )^{2}$ where *x*_*v*,*c*_ is the electrical percolation threshold. Fitting equation in Fig. 7 leads to $\sigma \propto (x_{v} {-}x_{v,c} )^{3}$ with *x*_*v*,*c*_ = 2.4 %. Exponent *t* = 3 exceeds the theoretically predicted value of 2 which is an indication of non-statistical ordered distribution of the conductive phase (i.e. clusters).

Thus, higher exponent means that higher charge current may develop. We believe that the current flows mainly along clusters and increases the ohmic character. The current–voltage characteristic (inset Fig. 6) measured on samples B showed voltage threshold at ~20 V. This proves that percolation sites exist, most probably between clusters.

Finally when combined with *M*_*s*_, the constitutive law remains a power-function of *σ* with 0.2 exponent. This gives the boundary of this work indicating a preferential increase in *σ* before *M*_*s*_. Scheme 2 is aimed to produce less clusterized films and would give a more favorable pattern (i.e. lower *t*). At this stage, the combination of *M*_*s*_ of 0.6–0.9 T with *σ* lower than 1 S/m with scheme 1 is unique.

**Fig. 8:**
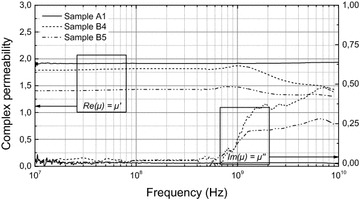
Complex permeability of samples A1, B4 and B5

To confirm the ability of the films for RF, measurements were carried out on samples A1, B4 and B5 (Fig. 8). Results for samples of lower *x*_*v*_ are not presented (uncertain). Overall, the real part *µ*′ and the imaginary *µ*″ are nearly constant up to 0.8 GHz with extremely low losses proving that the achieved low percolation conductivity was decisive. With A1 these characteristics extend to 10 GHz that is entirely unique. There is no sign of ferromagnetic resonance indicating that magnetocrystalline anisotropy of the particles was efficiently randomized. This remarkable feature greatly exceeds that of unsaturated magnetic materials (including ferrites) and recent works on ferrites nanocomposites.

(Castel et al. 2007), printable Co nanoparticles materials (Nelo et al. 2010) and Co epoxy-based nanocomposites (Raj et al. 2014). It proves that eddy currents are canceled and ferromagnetic losses are shifted until unconventional frequencies. In more details (at 1 GHz), *µ’* is 1.92 for A1 (19.3 %), 1.49 for B4 (18.1 %) and 1.87 for B5 (23.2 %). At first sight, *µ’* increases with *x*_*v*_. Considering the losses, *µ*″ is 1.26 × 10^−3^ for A1 (10^−1^ S/m) and ~1.3 × 10^−1^ for B4 (5 × 10^−3^ S/m) and B5 (4 × 10^−1^ S/m). There is no clear correlation with *σ*. The increase of losses around 1 GHz with series-B cannot be simply linked to eddy current but also originates from magnetic domain reversal and wall propagation that may be more dissipative as the structure is imperfect. Better properties may be expected with scheme 2 as residual clusters can be further reduced.

## Conclusion

The fabrication process of non-conductive ferromagnetic films based on metal-polymer nanocomposites was described. A core double-shell structure of cobalt–graphene–PS is reported able to preserve the net-moment of cobalt and to ensure electrical insulation. A thin shell of PS (~4 nm) was successfully grafted leading to a well-cohesive structure and preserving a high maximum filling ratio (~30 %). The use of radicals was shown more critical, however dispersion might be improved. The percolation conductivity was discussed. Despite unfavorable percolation-pattern (clusters), we achieved low conductivity (<1 S/m) leading to extremely low losses (10^−3^) at high frequency. To conclude we attested to the remarkable ability of such films for RF applications with a magnetization up to 0.6–0.9 T.
